# The effect of mouse strain on herpes simplex virus type 1 (HSV-1) infection of the central nervous system (CNS)

**DOI:** 10.1186/2042-4280-3-4

**Published:** 2012-03-26

**Authors:** Lorne F Kastrukoff, Allen S Lau, Eva E Thomas

**Affiliations:** 1 Department of Medicine, University of British Columbia, Vancouver V6T 1Z3, Canada; 2 Department of Pathology, British Columbia's Children's Hospital, Vancouver V6H 3 V4, Canada

**Keywords:** Herpes simplex virus 1(HSV-1), Central nervous system (CNS) infection, Mouse strain, dependent effect, HSV-1 induced CNS demyelination

## Abstract

**Background:**

Mice infected with HSV-1 can develop lethal encephalitis or virus induced CNS demyelination. Multiple factors affect outcome including route of infection, virus and mouse strain. When infected with a sub-lethal dose of HSV-1 strain 2 via the oral mucosa, susceptible SJL/J, A/J, and PL/J mice develop demyelinating lesions throughout the brain. In contrast, lesions are restricted to the brainstem (BST) in moderately resistant BALB/c mice and are absent in resistant BL/6 mice. The reasons for the strain differences are unknown.

**Methods:**

In this study, we combine histology, immunohistochemistry, and in-situ hybridization to investigate the relationship between virus and the development of lesions during the early stage (< 24 days PI) of demyelination in different strains of mice.

**Results:**

Initially, viral DNA and antigen positive cells appear sequentially in non-contiguous areas throughout the brains of BALB/c, SJL/J, A/J, and PL/J mice but are restricted to an area of the BST of BL/6 mice. In SJL/J, A/J, and PL/J mice, this is followed by the development of 'focal' areas of virus infected neuronal and non-neuronal cells throughout the brain. The 'focal' areas follow a hierarchical order and co-localize with developing demyelinating lesions. When antigen is cleared, viral DNA positive cells can remain in areas of demyelination; consistent with a latent infection. In contrast, 'focal' areas are restricted to the BST of BALB/c mice and do not occur in BL/6 mice.

**Conclusions:**

The results of this study indicate that susceptible mouse strains, infected with HSV-1 via the oral mucosa, develop CNS demyelination during the first 24 days PI in several stages. These include: the initial spread of virus and infection of cells in non-contiguous areas throughout the brain, the development of 'focal' areas of virus infected neuronal and non-neuronal cells, the co-localization of 'focal' areas with developing demyelinating lesions, and latent infection in a number of the lesions. In contrast, the limited demyelination that develops in BALB/c and the lack of demyelination in BL/6 mice correlates with the limited or lack of 'focal' areas of virus infected neuronal and non-neuronal cells in these two strains.

## Background

HSV-1 is a common infection in developed countries where rates of seropositivity usually exceed 50% [1,2]. In both humans and experimental animals, primary infection of the skin or mucosa results in the local replication of virus, infection of sensory nerve endings, and spread via retrograde axonal transport to the ganglia of the peripheral nervous system (PNS) where a productive infection of neurons ensues [1,2]. Although infectious virus is eventually cleared, a latent infection is established in neurons of the PNS ganglia [3,4].

HSV-1infection of the CNS is more complex with virus transmitted across synapses during primary infection and the development of latent infection in the brains of both humans [5-7] and experimental animals [8-10]. In humans, HSV-1 is a common cause of sporadic viral encephalitis [11,12] with mortality rates reaching 20-30% despite treatment [13]. Mice infected with HSV-1 can also develop lethal encephalitis with resistance to mortality being mouse strain dependent [14,15]. Further, HSV-1 is implicated in the development of CNS demyelinating disease in humans but its' role remains controversial [16-20]. Although a high incidence of HSV-1 in the brains and active plaques of MS patients is reported [21,22], virus is also present in controls. Recent studies, however, report an increased risk of MS in HSV-1 infected individuals without the DRB1*15 allele [23]; raising the possibility that this virus may play a role in the development of MS in individuals with a specific genotype. HSV-1 can also induce CNS demyelination in mice with the nature of the demyelinating lesions reported to be dependent on virus strain [24-31], route of infection [32], and mouse strain [33,34]. The mechanisms mediating the mouse strain effect are largely unknown. In this study, we combine histology, immunohistochemistry, and in-situ hybridization to investigate the relationship between virus and the development of lesions during the early stage (< 24 days PI) of demyelination in different strains of mice.

## Methods

### Mice

Inbred 8-10 week ♀ BL/6, BALB/c, SJL/J, A/J, and PL/J mice were purchased from the Jackson Laboratory, Bar Harbor, ME. Mice were housed in animal facilities of the Faculty of Medicine, University of British Columbia (UBC), and infected at 10 to 12 weeks of age. Principles of animal care (NIH publication No. 86-23, revised 1985) were followed in these studies along with the guidelines of the Institutional Animal Care and Use Committee of UBC.

### Virus and cells

HSV-1 (strain 2) was grown on BHK-21 cells with viral titers determined by plaque assay [35]. This strain of HSV-1 was isolated from human trigeminal ganglia, plaque purified, and characterized by Dr. Moira Brown (MRC Institute for Virology, Glasgow) [35,36]. The strain was selected from a large number of laboratory and clinical isolates because of the ability to induce CNS demyelination. Virus was stored at -80°C until used. The oral mucosa was inoculated with a sub-lethal dose, 2 × 10^5 ^plaque forming units (PFU) of virus, or mock infected using a scarification method previously described [33].

### Histology

The brains of three mice of each strain were removed at necropsy every 3 days PI and up to 30 days post-infection (PI). Additional mice were examined on intervening days as necessary.

Mice were perfused in-vivo with 4% paraformaldehyde in phosphate buffered saline (PBS). CNS tissue was dehydrated in alcohol and toluol, embedded in paraffin, and serially sectioned. Six micron thick coronal sections of the cerebral hemispheres (CR) along with transverse sections of the BST and cerebellum (CB) were made. Sections were counter stained with either hematoxylin-eosin (H & E), cresyl fast violet (CFV), or Luxol fast blue-cresyl fast violet (LFB-CFV). Sections were coded and examined in a blinded fashion with an Olympus BHS microscope.

### Immunohistochemistry (IHC)

Serial sections of the CR and BST-CB were examined by PAP IHC. Detection of HSV viral antigens employed polyclonal rabbit anti-HSV-1 antisera (B0114) that recognizes all viral proteins (DAKO, Burlingham, ON). Sections were pretreated with 0.5% hydrogen peroxide and washed in 0.05 M Tris-saline (pH7.6) plus 1% normal goat serum prior to incubation with anti-sera. This was followed by treatment with goat anti-rabbit IgG, rabbit PAP, and 0.03% 3-3' diaminobenzydine. Sections were counterstained with either CFV or LFB-CFV.

### In-situ hybridization (ISH)

Serial sections of the CR and BST-CB were examined by ISH. Sections were mounted on glass slides, deparaffinated, and rehydrated. Tissue was treated with 0.02 M HCL, washed, and treated with 0.01% Triton X-100 in PBS. Washed sections were treated with pronase (2.0 mg/ml in 50 mM Tris-HCl, pH 7.4), postfixed in 4% paraformaldehyde in PBS, treated with 100 mM triethanlamine (pH 8.0) plus 25 mM acetic anhydride and dehydrated in ethanol. Sections were then treated with a prehybridization mixture (2x SSC, formamide, Denhardt's solution, salmon sperm DNA, dextran sulfate) for 20 min at room temperature (RT). This was followed by treatment with a hybridization mixture (2x SSC, dextran sulfate, formamide, salmon sperm DNA, DTT, Denhardt's solution, ^35^S-labelled HSV cDNA) at 90°C for 7 min followed by 37°C for 16-24 hrs. The HSV cDNA probe was a 15 kb fragment (fragment G) derived from HSV-1 strain F and cloned in pTZ18. Specific activity of the probe was 2-7 × 10^8 ^cpm/μg of DNA. The fragment was previously determined not to cross react with cellular DNA. The tissue was washed, dehydrated in ethanol with 0.3 M ammonium acetate, air dried and coated with NTB-2 emulsion. The slides were stored at 4°C for two weeks, developed, and counterstained with H & E.

### Statistics

All analyses for statistically significant differences were performed with Student's *t *test. *P *< 0.05 is considered significant.

## Results

Three HSV-1 infected mice of each strain (BL/6, BALB/c, SJL/J, A/J, and PL/J) were examined at necropsy every 3 days from day 0 through 30 PI. In some cases, additional mice were examined on intervening days along with mock infected controls. CR and BST-CB were serially sectioned and yielded ~300 sections of the CR and ~140 sections of the BST-CB per mouse. The serial sections were examined for viral DNA by ISH with H&E counterstain, for viral antigen by IHC and CFV counterstain, or both viral antigen and demyelination by IHC with LFB-CFV counterstain. None of the mock infected controls were positive for HSV-1 DNA, viral antigen, or CNS demyelination.

### In BL/6 mice, HSV-1 DNA and antigen positive cells are restricted to the BST

**Figure 1:**
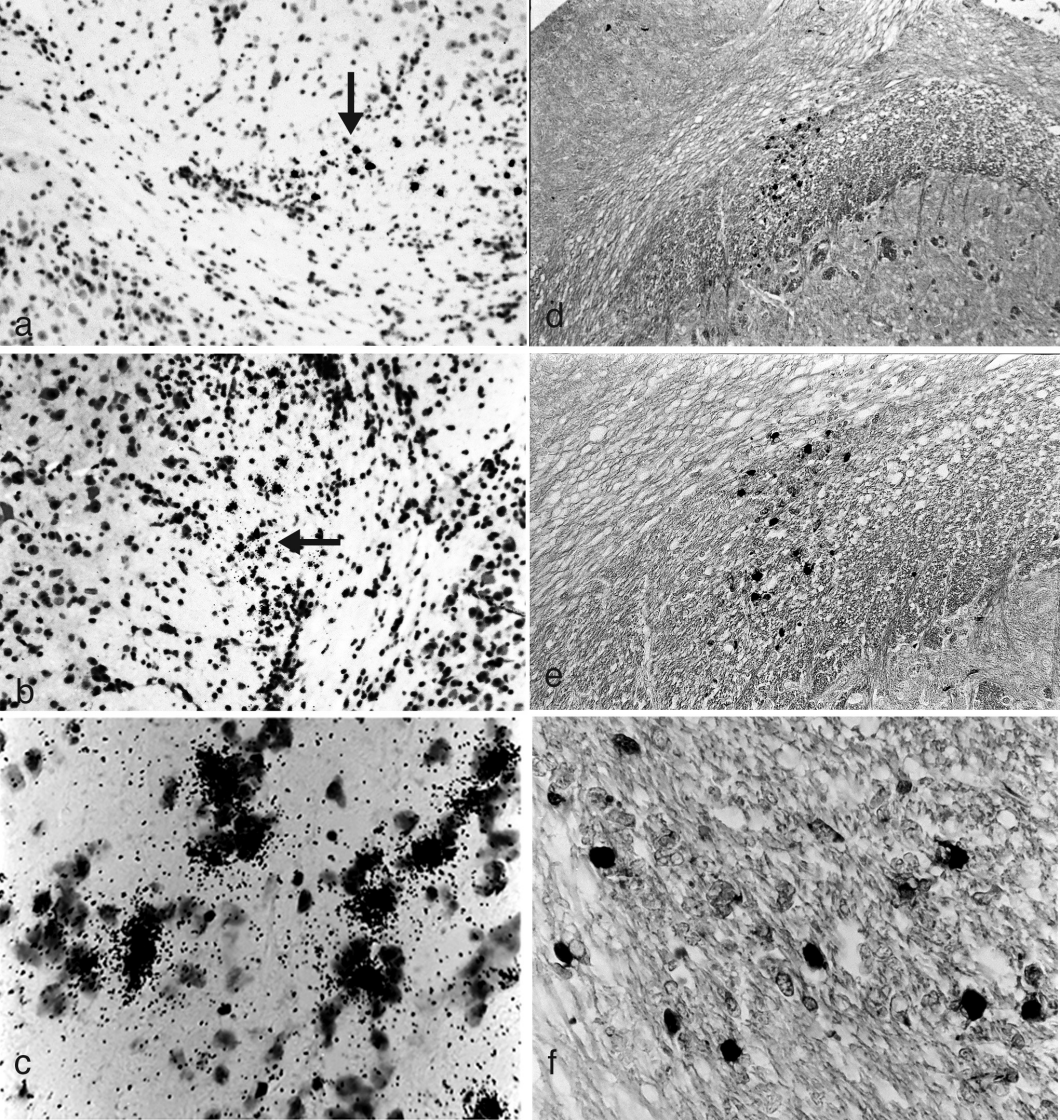
**HSV-1 DNA and antigen positive cells are not present diffusely throughout the BST of BL/6 mice but are restricted to an area associated with the RTNBST**. Ten-12 week ♀ BL/6 mice were infected with HSV-1 strain 2 via the oral mucosa. Three mice were examined every 3 days at necropsy from day 0 through 30 PI. Six micron thick serial sections of the brain were examined for ^35^S-labelled HSV DNA (ISH) and viral antigens (IHC) and counterstained with either H & E or CFV respectively. In representative sections, both viral DNA (Figure 1 a - c) and viral antigen (Figure 1 d - f) are identified on day 3 PI and restricted to a small area of the BST associated with the roots of the trigeminal nerve (RTNBST). (**a**) Small area of ^35^S-labelled HSV DNA positive cells (arrow) in the BST on day 3 PI (x100) (**b**) Higher power photomicrograph of HSV-1 DNA positive cells (arrow) on day 3 PI (x200) (**c**) High power photomicrograph of HSV-1 DNA positive cells on day 3 PI (x400) (**d**) Small area of HSV-1 antigen positive cells in the BST on day 3 PI (x100) (**e) **Higher power photomicrograph of HSV-1 antigen positive cells on day 3 PI (x200) (**f) **High power photomicrograph of HSV-1 antigen positive cells on day 3 PI (x400).

In BL/6 mice infected with HSV-1 strain 2 via the oral mucosa, the spread of infectious virus is restricted to the BST [37] and CNS demyelinating lesions do not occur [33]. To further examine the location of viral DNA and antigen in the brain, HSV-1 infected 10-12 week ♀ BL/6 mice were examined every 3 days from day 0 through 30 PI. Viral DNA and antigen positive cells cannot be identified throughout the brain nor are they present diffusely throughout the BST (data not shown). Viral DNA (Figure 1a-c) and antigen (Figure 1d-f) positive cells are restricted to a small area of the BST in relation to the roots of the trigeminal nerve (RTNBST). The appearance and clearance of viral antigen in the brains of BL/6 mice is 3 and 12 days PI respectively (Table 1) but viral DNA positive cells can remain for at least 30 days PI (data not shown).

**Table 1:** The appearance and clearance of HSV-1 antigen positive cells and 'focal' areas of viral antigen positive cells throughout the brains of inbred strains of mice

Mouse strain^a^	Initial appearance of viral antigen positive cells throughout the brains				Clearance of viral antigen throughout the brain (days PI)
	RTNBST^b^	BST^c^	CB^d^	CR^e ^(days PI)	

BL/6	3	-	-	-	12

BALB/c	3	3	6	7	18

SJL/J	6	6	7	8	21

A/J	3	3	6	7	21

PL/J	3	3	5	6	21




**Mouse strain^a^ ext**	**Initial appearance of 'focal' areas of viral antigen positive cells throughout the brain**				**Clearance of viral antigen throughout the brain (days PI)**

	RTNBST^a^	BST^b^	CB^c^	CR^d ^(days PI)	

BL/6	-	-	-	-	12

BALB/c	5	-	-	-	18

SJL/J	9	9	12	15	21

A/J	5	5	9	12	21

PL/J	5	5	6	9	21

^a^Ten-12 week ♀ inbred mice were infected with HSV-1 strain 2 via the oral mucosa. Three mice of each strain were examined every 3 days or as necessary from Day 0 through 30 PI.

^b^Roots of the trigeminal nerve in the brainstem

^c^Brainstem

^d^Cerebellum

^e^Cerebral hemispheres

### In BALB/c mice, HSV-1 antigen positive cells appear sequentially in non-contiguous areas throughout the brain

**Figure 2:**
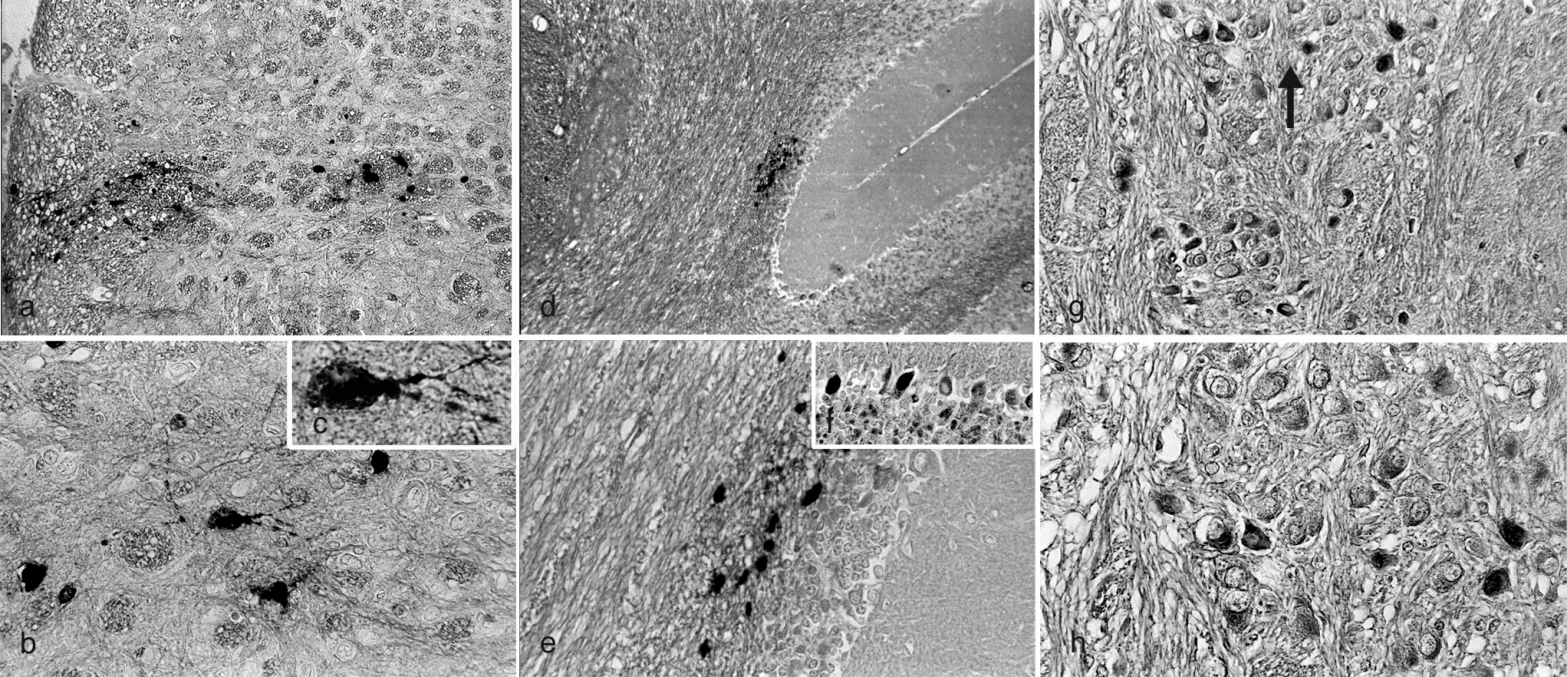
**HSV-1 antigen positive cells appear sequentially in non-contiguous areas throughout the brains of BALB/c mice**. Ten-12 week ♀ BALB/c mice were infected with HSV-1 strain 2 via the oral mucosa. Three mice were examined every 3 days at necropsy from day 0 through day 30 PI. Serial sections of the brain were examined for viral antigens (IHC) and counterstained with CFV. In representative sections, viral antigen was first identified in the BST on day 3 PI. **(a) **Viral antigen positive cells in the BST, 3 days PI (x100) **(b) **Higher power photomicrograph of HSV-1 antigen positive cells on day 3 PI (x400) (**c**) High power photomicrograph of HSV-1 antigen positive cell with the morphology of a neuron on day 3 PI (x1000). Viral antigen was first identified in the cerebellum (CB) on day 6 PI (**d**) Viral antigen positive cells in the CB, 6 days PI (x100) (**e) **Higher power photomicrograph of HSV-1 antigen positive cells on day 6 PI (x400) (**f**) High power photomicrograph of HSV-1 antigen positive Purkinje cells in the CB, 6 days PI (x400). Viral antigen was first identified in the cerebrum (CR) on day 7 PI (**g**) Viral antigen positive cells (arrow) in the CR, 7 days PI (x200) (**h**) Higher power photomicrograph of HSV-1 antigen positive cells with the morphology of neurons in the CR, 7 days PI (X400).

In BALB/c mice, infected with HSV-1 strain 2 via the oral mucosa, virus spreads throughout the brain [37] but CNS demyelinating lesions are restricted to the trigeminal root entry zone of the BST [33]. To identify viral antigen in the brain, HSV-1 infected 10-12 week ♀ BALB/c mice were examined every 3 days from day 0 through 30 PI. In contrast to BL/6 mice, viral antigen positive cells in BALB/c mice appear sequentially in non-contiguous areas throughout the brain. Antigen positive cells first appear in the BST on day 3 PI (Figure 2a &2b), in the CB on day 6 PI (Figure 2d &2e), and in the CR on day 7 PI (Figure 2g). Many of the cells have the morphology of neurons (Figure 2c, 2f, &2h). Viral antigen positive cells also appear sequentially in non-contiguous areas throughout the brains of SJL/J, A/J, and PL/J mice (data not shown). In BALB/c mice, 'focal' areas of viral antigen positive neuronal and non-neuronal cells are restricted to an area in relation to the RTNBST (data not shown). The appearance and clearance of viral antigen in the brains of BALB/c mice is 3 and 18 days PI respectively (Table 1) but viral DNA positive cells can remain for at least 30 days PI (data not shown).

### In SJL/J, A/J, and PL/J mice, 'focal' areas of viral antigen positive neuronal and non-neuronal cells appear sequentially in non-contiguous areas throughout the brain

**Figure 3:**
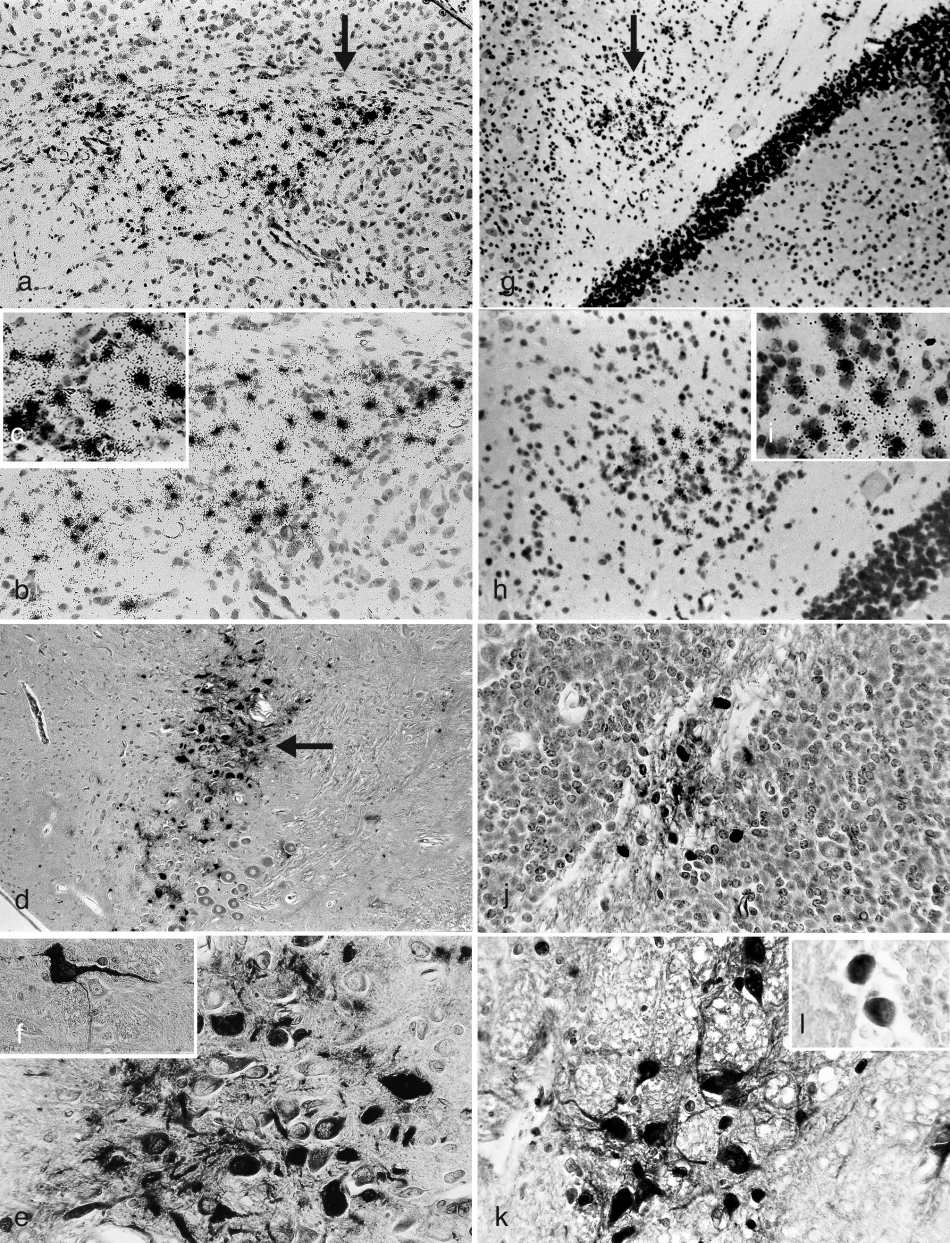
**'Focal' areas of HSV-1 DNA and antigen positive cells develop sequentially in non-contiguous areas of the BST and CB of PL/J mice**. Brains from 10-12 week ♀ PL/J mice, infected with HSV-1 via the oral mucosa, were examined for viral DNA and antigen. Serial sections of the brain were examined for ^35^S-labelled HSV DNA (ISH) and viral antigens (IHC) and counterstained with either H & E or CFV respectively. **'**Focal' areas of ^35^S-labelled HSV-1 DNA (Figure 3 a-c) and viral antigen (Figure 3 d-f) positive cells are identified in the BST **(a) **A 'focal' area of HSV-1 DNA positive cells in the BST (arrow), 3 days PI (x100) **(b) **Higher power photomicrograph, 3 days PI (x 200) **(c) **High power photomicrograph, 3 days PI (x400) **(d) **A 'focal' area of viral antigen positive cells in the BST (arrow), 5 days PI (x100) **(e) **Higher power photomicrograph, 5 days PI (x400) **(f) **Higher power photomicrograph of viral antigen positive neuron, 5 days PI (x400). 'Focal' areas of ^35^S-labelled HSV-1 DNA (Figure 3g-i) and viral antigen (Figure 3 j-l) positive cells are identified in the CB. **(g) **A 'focal' area of HSV-1 DNA positive cells in the CB (arrow), 5 days PI (x100) **(h) **Higher power photomicrograph, 5 days PI (x200) **(i) **High power photomicrograph, 5 days PI (x400) **(j) **A 'focal' area of viral antigen positive cells in the CB, 6 days PI (x200) **(k) **Higher power photomicrograph, 6 days PI (x400) **(l) **Higher power photomicrograph of viral antigen positive Purkinje cells, 6 days PI (x400).

In SJL/J, A/J, and PL/J mice, infected with HSV-1 strain 2 via the oral mucosa, infectious virus spreads sequentially throughout the brain [33] and is associated with the development of early stage (< 24 days PI) CNS demyelination throughout the brain [34]. To identify 'focal' areas of viral DNA and antigen positive neuronal and non-neuronal cells throughout the brain, HSV-1 infected 10-12 week ♀ mice of all three strains were examined every three days from day 0 through 30 PI. In PL/J mice, 'focal' areas of HSV-1 DNA positive cells appear sequentially in non-contiguous areas throughout the brain. These areas are shown in representative sections of the BST (Figure 3a-c) and CB (Figure 3g-i). 'Focal' areas of viral antigen positive cells in PL/J mice also appear sequentially in non-contiguous areas throughout the brain. These areas are shown in representative sections of BST (figure 3d-f) and CB (Figure 3j-l). 'Focal' areas of HSV-1 DNA positive and antigen positive cells also develop throughout the brains of SJL/J and A/J mice (data not shown). The appearance and clearance of the 'focal' areas are mouse strain dependent (Table 1).

### Number and size of the 'focal' areas of viral antigen positive cells define a hierarchy among different strains of mice

**Figure 4:**
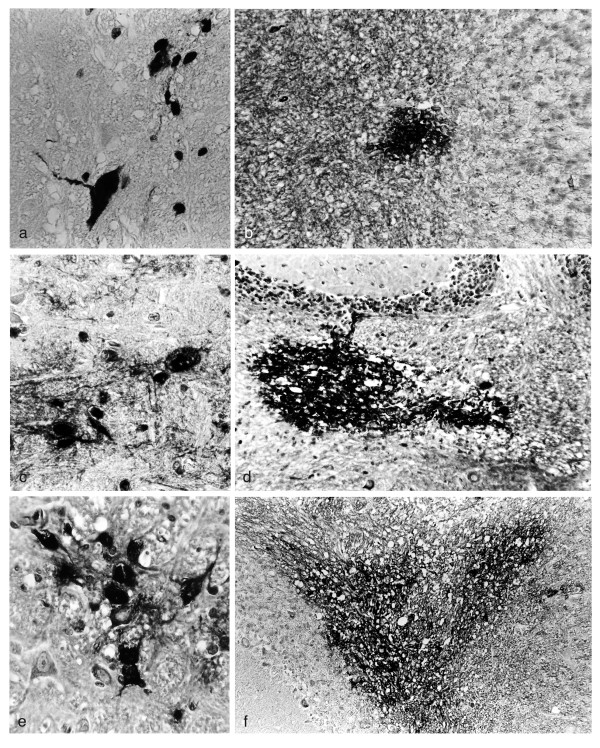
**A hierarchy exists among 'focal' areas of HSV-1 antigen positive cells in SJL/J, A/J, and PL/J mice**. 'Focal' areas of viral antigen positive cells in serial sections of the BST-CB derived from 3 mice of each strain on day 6, 9, 12, and 15 PI were examined. Serial sections were examined by IHC and the number of 'focal' areas of viral antigen in the BST and CB per slide determined. As one 'focal' area could be present in several serial sections, the greatest diameter of the lesion at a magnification of 400x for the BST and 200x for the CB was recorded. Representative 'focal' areas of HSV-1 antigen positive cells in the BST-CB of virus infected 10-12 week ♀ mice are shown. Representative 'focal' areas in the BST of **(a) **SJL/J, 9 days PI (x400) **(c) **A/J, 6 days PI (x400) and **(e) **PL/J, 6 days PI (x400) mice are shown. Representative 'focal' areas in the CB of **(b) **SJL/J, 12 days PI (x200) **(d) **A/J, 9 days PI (x200) and **(f) **PL/J, 6 days PI (x200) mice are also shown.

The number and size of CNS demyelinating lesions define a hierarchical order among different strains of mice infected with HSV-1 strain 2 via the oral mucosa [34]. To identify the number and size of 'focal' areas of viral antigen positive cells in the brain, HSV-1 infected 10-12 week ♀ SJL/J, A/J, and PL/J mice were examined every 3 days from day 0 through 30 PI. In SJL/J mice, 'focal' areas are few in number and small in size (Figure 4a &4b) while in PL/J mice the areas are greater in number and larger in size (Figure 4e &4f). In A/J mice the number and size of the 'focal' areas are intermediate between SJ/L and Pl/J mice (Figure 4c &4d). The number and size of the 'focal' areas also form a hierarchy among the mouse strains.

The number and size of the 'focal' areas of viral antigen positive cells were determined in every fourth section of the 140 serial BST-CB sections obtained from each of three mice of the three strains. To allow a comparison between the three mouse strains, IHC sections counterstained with CFV were examined on the first day the 'focal' areas appeared in the BST (day 9 PI in SJL/J mice and day 5 PI in A/J and PL/J mice) and the CB (day 12 PI in SJL/J mice, day 9 PI in A/J mice, and day 6 PI in PL/J mice) (Table 2). Differences in the number and size of the 'focal' areas among the three strains were statistically significant (Table 2).

**Table 2:** The number and size of 'focal' areas of HSV-1 antigen positive cells in the BST-CB of inbred strains of mice

Mouse strain^a^	BST^b ^examined for focal' areas on day (PI)	CB^c ^examined for 'focal' areas on day (PI)	# of 'focal' areas per section^d^		Size of 'focal' area^e ^(μm)	
SJL/J	9	12	0.6 ± 0.7	0.7 ± 0.7	97 ± 8	153 ± 6

A/J	5	9	0.7 ± 0.8	2.6 ± 1.0	156 ± 8	285 ± 9

PL/J	5	6	1.6 ± 1.0	4.6 ± 1.7	207 ± 15	373 ± 21

^a^Three 10-12 week ♀ mice of each strain were infected with HSV-1 strain 2 via the oral mucosa. 'Focal' areas of viral antigen positive cells were determined in serial sections of the brainstem-cerebellum (BST-CB) by IHC and CFV counterstain.

^b^Sections of the BST were examined on the first day that 'focal' areas appear for each mouse strain.

^c^Sections of the CB were examined on the first day that 'focal' areas appear for each mouse strain.

^d^Average number of 'focal' areas of viral antigen ± SD per section. Differences among mouse strains are all statistically significant at P < 0.001 except for the difference between SJL/J and A/J BST which was NS.

^e^As a single 'focal' area could be represented in several sections, the greatest diameter for each'focal' area was determined. The average diameter ± SD of the 'focal' areas for the three mouse strains was then calculated. Differences between the three mouse strains are all statistically significant at P < 0.001.

### Demyelinating lesions co-localize with 'focal' areas of HSV-1 antigen positive cells throughout the brains of SJL/J, A/J, and PL/J mice

**Figure 5:**
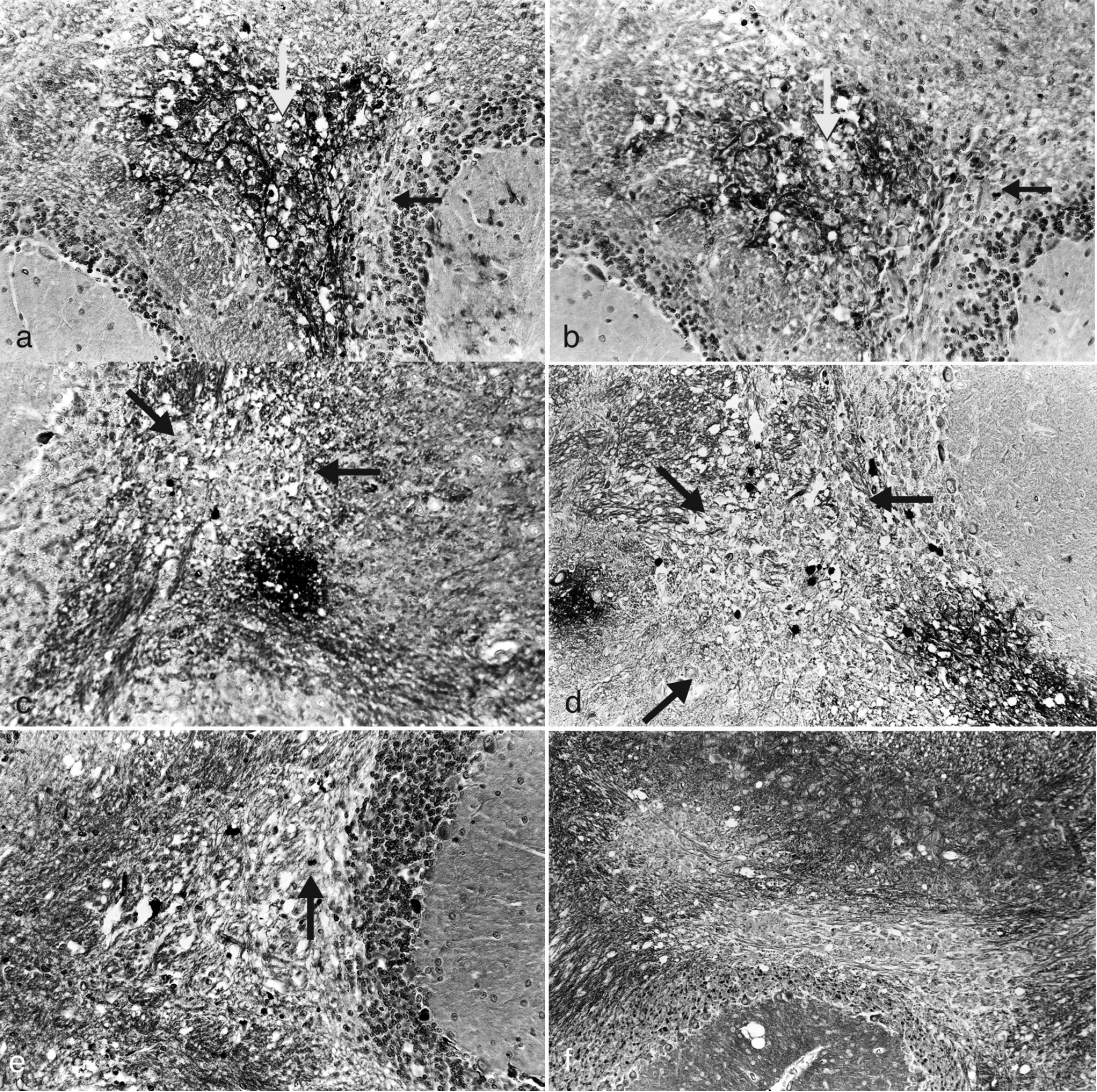
**'Focal' areas of HSV-1 antigen positive cells and demyelinating lesions co-localize throughout the brains of SJL/J, A/J, and PL/J mice**. Serial IHC sections counterstained with LFB-CFV were examined for 'focal' areas of viral antigen positive cells and CNS demyelination in the BST and CB. 'Focal' areas and demyelinating lesions co-localize throughout the brains of 10-12 week ♀ SJL/J, A/J, and PL/J mice infected with HSV-1 via the oral mucosa. Initially, island of demyelination are identified within or adjacent to 'focal' areas of viral antigen. Representative sections from the CB of PL/J mice identify these areas within (white arrow) or adjacent to (black arrow) 'focal' areas of viral antigen **(a) **8 days PI (x200) and **(b) **9 days PI (x200). As the 'focal' areas of viral antigen are cleared, the demyelinating lesions increase in size. Representative sections from the CB of SJL/J mice identify areas of demyelination (arrows) adjacent to 'focal' areas of viral antigen **(c) **14 days PI (x200) and **(d) **15 days PI (x200). Although individual antigen positive cells can remain in the demyelinating lesions for a period of time **(e) **antigen positive cell (arrow) in the CB of PL/J mouse, 18 days PI (x200), 'focal' areas of viral antigen are eventually cleared **(f) **demyelinating lesions in the CB of PL/J mouse, 20 days PI (x200).

The relationship between 'focal' areas of HSV-1 antigen positive neuronal and non-neuronal cells and demyelinating lesions throughout the brain was determined by IHC in sections counterstained with LFV-CFV. HSV-1 infected 10-12 week ♀ SJL/J, A/J, and PL/J mice were examined every 3 days from day 0 through 30 PI. Initially, islands of demyelination develop either adjacent to or within 'focal' areas of viral antigen positive cells (Figure 5a &5b). As viral antigen is cleared, the 'focal' areas decrease in size while the demyelinating lesions increase in size (Figure 5c &5d). Further, viral antigen positive cells in the demyelinating lesions decrease in number and are eventually cleared (Figure 5e &5f). Viral antigen is cleared throughout the brain in all mouse strains by day 21 PI (Table 1).

The relationship between 'focal' areas of HSV-1 antigen positive cells and demyelinating lesions was determined in every fourth section of the 140 serial BST-CB sections obtained from each of three mice of the three strains. To allow a comparison between the three mouse strains, IHC sections counterstained with LFB-CFV were examined on the first day demyelinating lesions appeared in the BST (day 12 PI in SJL/J mice and day 6 PI in A/J and PL/J mice) and the CB (day 15 PI in SJL/J mice, day 12 PI in A/J mice, and day 9 PI in PL/J mice) (Table 3). Although the number of 'focal' areas of antigen positive cells and demyelinating lesions is mouse strain dependent (Table 3), in all mouse strains, the percentage of demyelinating lesions that co-localize with 'focal' areas of viral antigen is greater than 90%. Not all 'focal' areas of viral antigen co-localized with demyelinating lesions. Either the demyelinating lesions have yet to develop in relation to the 'focal' areas of viral antigen positive cells or not all 'focal' areas are associated with demyelinating lesions.

**Table 3:** The relationship between 'focal' areas of HSV-1 antigen and demyelinating lesions in the BST-CB of inbred strains of mice

Mouse strain^a^	BST^b ^on day (PI)	CB^c ^on day (PI)		# of 'focal' areas^d^	# of lesions^e^		% of 'focal' areas that co-localize with lesions	
			BST	CB	BST	CB	BST	CB

SJL/J	12	15	19.7 ± 1.5	25.5 ± 4.1	18.2 ± 1.0	24.0 ± 3.7	92	94

A/J	6	12	24.8 ± 2.0	93.7 ± 1.1	24.1 ± 1.8	90.6 ± 1.0	97	97

PL/J	6	9	56.7 ± 3.1	163.7 ± 7.4	53.4 ± 2.1	162.4 ± 6.0	94	99

^a^Three 10-12 week ♀ mice of each strain were infected with HSV-1 strain 2 via the oral mucosa. 'Focal' areas of viral antigen positive cells and demyelinating lesions were determined in serial sections of the brainstem-cerebellum (BST-CB) by IHC and LFB-CFV counterstain.

^b^Both 'focal' areas of viral antigen and demyelinating lesions were determined in the BST of all three mouse strains. Sections of the BST were obtained on the first day demyelinating lesions appear in that mouse strain [24].

^c^Both 'focal' areas of viral antigen and demyelinating lesions were determined in the CB of all three mouse strains. Sections of the CB were obtained on the first day demyelinating lesions appear in that mouse strain [24].

^d^Average number of 'focal' areas of viral antigen ± SD in the BST and CB of each of three mice. Differences among mouse strains are all statistically significant at P < 0.005.

^e^Average number of demyelinating lesions ± SD among in the BST and CB of each of three mice. Differences among mouse strains are all statistically significant at P < 0.005.

### Despite the clearance of viral antigen positive cells, HSV-1 DNA positive cells can remain in demyelinating lesions of SJL/J, A/J, and PL/J mice

**Figure 6:**
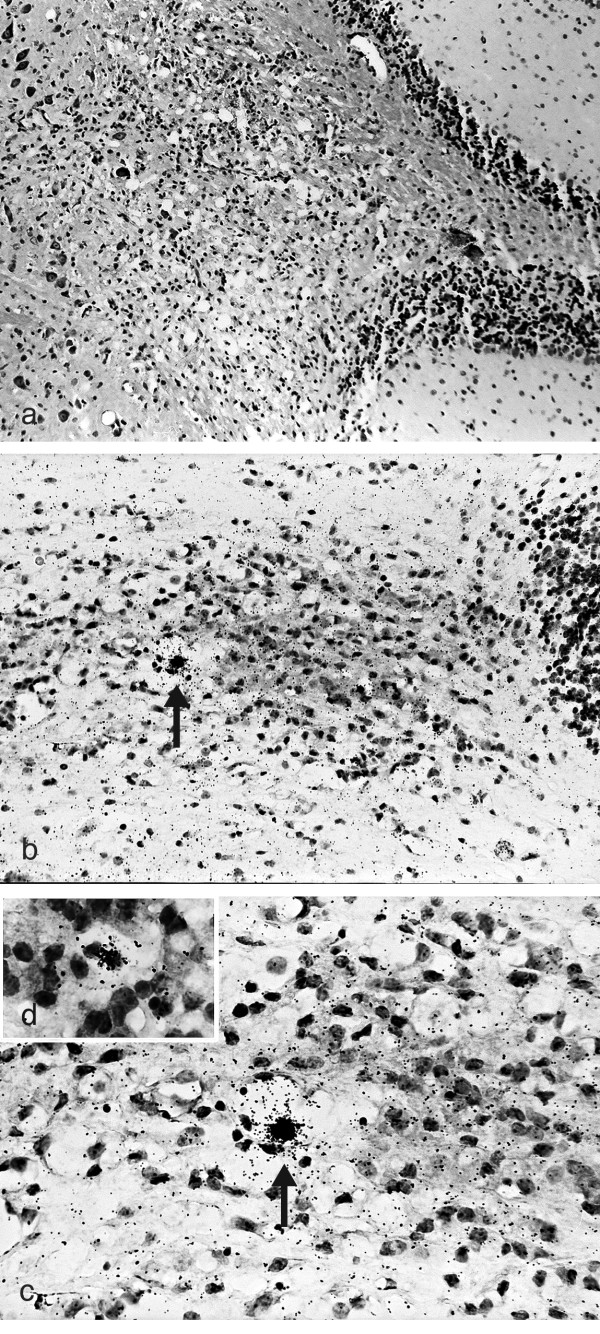
**HSV-1 DNA positive cells remain in an area of demyelination following the clearance of viral antigen**. ^35^S-labelled HSV-1 DNA positive cells can remain in demyelinating lesions of 10-12 week ♀SJL/J, A/J, and PL/J mice infected with virus via the oral mucosa. Adjacent serial sections were examined by ISH and histology. In representative sections from the CB of HSV-1 infected PL/J mice **(a) **demyelinating lesion are identified, 26 days PI, (x100), LFB-CFV while adjacent sections identify **(b) **HSV-1 DNA positive cells (arrow), 26 days PI, (x200), ISH counterstained with H & E. **(c) **Higher power photo-micrograph of HSV-1 DNA positive cells (arrow), 26 days PI, (x400), ISH/H & E. **(d) **High power photo-micrograph of HSV-1 DNA positive cell, 26 days PI (x1000), ISH/H & E.

Infection of mice with HSV-1 can result in the development of viral latency in the CNS [8-10]. The relationship between ^35^S-labelled HSV-1 DNA positive cells and CNS demyelinating lesions was examined in adjacent serial sections of the BST-CB using histology and ISH. HSV-1 infected 10-12 week ♀ SJL/J, A/J, and PL/J mice were examined every 3 days from day 0 through 30 PI. Although infectious virus [34] and viral antigen (Table 1) are eventually cleared from the brains of all three mouse strains, the clearance of infectious virus precedes that of viral antigen. In contrast, HSV-1 DNA positive cells can remain in the brain beyond 25 days PI and are identified in demyelinating lesions of all three strains (Figure 6). This is consistent with latently infected cells remaining in an area of demyelination.

## Discussion

Mice infected with HSV-1 can develop CNS demyelinating lesions [24-32] but their development is determined by a number of factors including route of infection and virus strain. When infected in the pinna of the ear, BALB/cAJcl mice develop demyelination restricted to the descending root of the facial nerve [32]. When infected with the Rodanus strain of HSV-1, both Swiss and BALB/c mice develop demyelination restricted to the TREZ [24-27] but when infected with Roziman strain F, demyelination develops in both the TREZ and BST of the two mouse strains [28-31]. In contrast, BALB/c mice infected with a HSV-1 recombinant virus expressing IL-2 develop lesions in the optic nerve, brain, and spinal cord [38,39]. Further, BALB/c mice infected with strains McKrae or KOS [38-40] or BL/6 mice infected with strain McRae [40] do not develop CNS demyelination but macrophage depleted BALB/c and BL/6 mice, infected with strain KOS or McKrae respectively, do develop lesions throughout the CNS [40]. A number of studies performed by Ghiasi and colleagues have implicated CD4^+^CD25^+^FoxP_3_^+ ^T cells as playing a pathogenic role in the development of demyelinating lesions when Il-12 p70 macrophages are ablated [40].

Mouse strain can also influence the development of HSV-1 induced CNS demyelination. We previously reported that SJL/J, A/J, and PL/J but not BL/6 mice, infected with a sub-lethal dose of HSV-1 strain 2 via the oral mucosa, develop demyelinating lesions throughout the brain while lesions in BALB/c mice are restricted to the TREZ of the BST [33,34]. The lesions are characterized by demyelination, a mononuclear cell infiltrate, and relative preservation of axons [33,34,41]. Further, SJL/J, A/J, and PL/J mice develop lesions sequentially throughout the brain during the early stage (< 24 days PI), but randomly in both A/J and PL/J mice during the intermediate stage (1-3 months PI), and only in PL/J mice during the late stage (> 3 months PI) of demyelination [34]. During the early stage of demyelination, lesions are immune mediated [42], and their appearance correlates with the sequential spread of infectious virus throughout the brain [33,37]. The number and size of the lesions follow a hierarchical order among the mouse strains [33,34]. Although the influence of mouse strain on CNS infection is recognized and defined for a number of different viruses [43-46], our understanding of this effect on HSV-1 CNS infection, including the development of demyelination, is at a preliminary stage.

In this study we combine histology, IHC, and ISH to further define the effect of mouse strain on the development of early stage demyelination in mice infected with HSV-1 strain 2 via the oral mucosa.

Results of this study argue that HSV-1 induced CNS demyelination throughout the brain in susceptible strains of mice, develops in several stages. First, viral DNA and antigen positive cells appear sequentially throughout the brain, localize to non-contiguous areas of the brain, and primarily to cells with the morphology of neurons (Table 1). This is consistent with previous reports on the spread and localization of HSV-1 in the brain [47-49] resulting from transneuronal transport of virus from the periphery to the CNS and synaptically determined relationships in the CNS [30,49-52]. However, results of this study indicate that this does not occur in all mouse strains. Although it is the case in BALB/c, SJL/J, A/J, and PL/J mice, it is not in BL/6 mice where viral DNA and antigen are restricted to a specific area of the BST. The restriction of HSV-1 to the BST in this strain was previously identified by viral titration studies [37] but the results of this study indicate a far greater degree of restriction occurs in the BST then was previously recognized.

Second, 'focal' areas of viral antigen positive neuronal and non-neuronal cells develop throughout the brain (Table 1). The areas appear sequentially and localize to non-contiguous areas throughout the brain. Their development may result from the uptake and replication of HSV-1 in neurons resulting in the degeneration of infected cells, loss of cellular integrity, and release of virus [53-56]. Having a high affinity for herpes viruses [57-59], glia can become infected in areas surrounding the disintegrating neurons [28,31]. Further, the development of 'focal' areas of viral antigen with HSV-1 has similarities to other herpes virus infections including pseudorabies virus where reactive gliosis and macrophage infiltration provides a barrier to the diffusion of virus through the extra-cellular compartment by isolating and phagocytosing virus [60,61]. Results of our study identify 'focal' areas of viral antigen positive cells throughout the brains of SJL/J, A/J, and PL/J mice but not in BALB/c mice where the 'focal' areas are restricted to small areas of the BST. In BL/6 mice 'focal' areas of viral antigen positive cells do not develop in the brain. In the strains developing 'focal' areas of viral antigen positive cells throughout the brain, there is a hierarchical order for size and number. Lesions are numerous and large in PL/J mice but few in number and small in size in SJL/J mice. A/J mice are intermediate for both number and size. The differences are statistically significant (Table 2). Previous studies have attributed differences in mouse strain susceptibility to the extent of glial infection with HSV-1 [33,41,62,63] while other studies have reported differences in resistance to HSV-1to be mediated directly by glial cells [31,64,65]. A similar hierarchical order was previously identified for number and size of demyelinating lesions developing in the same strains of mice [34].

Third, 'focal' areas of viral antigen positive cells co-localize with demyelinating lesions that develop throughout the brain (Table 3). While the co-localization of 'focal' areas with demyelinating lesions occurs in SJL/J, A/J, and PL/J mice, they are restricted to a small area of the BST of BALB/c mice, and do not develop in BL/6 mice. Although the results argue that demyelinating lesions evolve from 'focal' areas of antigen positive cells, it is unclear if they develop from within or adjacent to the 'focal' areas.

Fourth, as 'focal' areas and individual viral antigen positive cells are cleared from the lesions, viral DNA positive cells, consistent with a latent infection, remain in the demyelinating lesions. Although viral latency in the CNS of mice is reported after infection with HSV-1 [8-10], the presence of latent virus within demyelinating lesions of mice has not been previously reported to our knowledge.

Based on these results, we argue that the effect of mouse strain on the development of CNS demyelination during the early stage of HSV-1infection (< 24 days PI) is determined by the ability of virus to spread through the PNS and CNS of a specific mouse strain and the ability of the host to mount an immune response that restricts viral spread and clears virus from the brain. In all mouse strains infected with HSV-1 via the oral mucosa, virus spreads via retrograde axonal transport to the TG of the PNS [1,2] and is followed by access to the CNS. In a number but not all mouse strains, virus spreads by transneuronal transport throughout the brain with the development of focal and non-contiguous neuronal infection determined by synaptically defined relationships in the CNS [30,49-52]. In many but not all mouse strains, this is followed by the development of 'focal' areas of antigen positive neuronal and non-neuronal cells that result from the replication of virus in neurons, degeneration of infected cells, release of virus [53-56], and subsequent infection of surrounding glia [57-59]. The hierarchical order of number and size of 'focal' areas reflects mouse strain differences in resistance of glia to HSV-1 [33,41,64,65]. An immune response clears 'focal' areas of viral antigen positive cells from the CNS but results in the development of demyelinating lesions [42]. In A/J, PL/J, and SJL/J mice, viral antigen appears early throughout the brain but viral clearance is delayed until day 21 PI (Table 1). A delay in the development of an immune response in these mouse strains could explain the delay in viral clearance and allow for the development of 'focal' areas of antigen positive neuronal and non-neuronal cells. In BL/6 mice, viral antigen also appears early in the CNS but in contrast to other mouse strains, virus is restricted to the BST. The restriction of viral spread and the failure to develop 'focal' areas of viral antigen positive cells is likely responsible for the absence of demyelinating lesions. Recently, we provided evidence that the restriction of viral spread in BL/6 mice results from redundancy in the immune system and mediated by NK/NKT and CD8^+ ^T-lymphocytes [37]. Further, clearance of viral antigen from the brains of BL/6 mice occurs on day 12 PI; earlier then occurs in other mouse strains (Table 1). The immune mechanisms mediating the clearance of virus has not yet been defined. In BALB/c mice, viral antigen also appears early throughout the brain (Table 1) but in this strain viral clearance occurs by day 18 PI. This is delayed compared to BL/6 mice but early when compared to SJL/J, A/J, and PL/J mice. The delay in clearance could explain the spread of virus throughout the brain but when compared to SJL/J, A/J, and PL/J mice, might be sufficient to clear virus before 'focal' areas of viral antigen positive cells develop.

## Conclusions

When infected with a sub-lethal dose of HSV-1 lab strain 2 via the oral mucosa, susceptible SJL/J, A/J, and PL/J mice develop demyelinating lesions throughout the brain. In contrast, in moderately resistant BALB/c mice, demyelinating lesions are restricted to the TREZ of the BST. Resistant BL/6 mice do not develop CNS demyelination. In this study, we combine histology, IHC, and ISH to further investigate the effect of mouse strain on the early stage of demyelination (< 24 days PI).

Results of this study indicate that demyelinating lesions throughout the brain of susceptible mice develop in several stages. Initially, viral DNA and antigen infected cells, largely neurons, appear in non-contiguous areas throughout the brain. This is followed by the development of 'focal' areas of viral antigen positive neuronal and non-neuronal cells in non-contiguous areas throughout the brain. The number and size of the 'focal' areas follow a hierarchical order among the different mouse strains. Next, the 'focal' areas of viral antigen positive cells are seen to co-localize with demyelinating lesions suggesting they evolve from the 'focal' areas. As viral antigen positive cells and 'focal' areas are cleared, viral DNA positive cells consistent with a latent infection can remain in the areas of demyelination. All of these stages occur in susceptible SJL/J, A/J, and PL/J mice but not in moderately resistant BALB/c mice where 'focal' areas of antigen positive cells are restricted to a small area of the BST, and not in resistant BL/6 mice where 'focal' areas do not develop in the brain.

We hypothesize that the development of demyelinating lesions throughout the brain of susceptible mouse strains results from the non-contiguous spread of virus throughout the brain and the inability of the host to either restrict viral spread or clear virus from the brain prior to the development of 'focal' areas of viral antigen positive neuronal and non-neuronal cells.

## Abbreviatons

BL/6: C57BL/6; BST: Brainstem; CB: Cerebellum; CNS: Central nervous system; CR: Cerebral hemispheres; HSV: Herpes simplex virus; HSV-1: Herpes simplex virus type 1; IHC: Immunohistochemistry; ISH: In-situ hybridization; MS: Multiple sclerosis; PAP: Peroxidase-antiperoxidase; PBS: Phosphate buffered saline; PCR: Polymerase chain reaction; PFU: Plaque forming units; PI: Post infection; PNS: Peripheral nervous system; RTNBST: Roots of the trigeminal nerve in the brainstem; TG: Trigeminal ganglia; TREZ: Trigeminal route entry zone

## Competing interests

The authors declare that they have no competing interests.

## Authors' contributions

ASL carried out the histology, immunohistochemistry, and in-situ hybridization studies. EET was a major participant in the design and co-ordination of this study. LFK conceived the study, participiated in its design and coordination and undertook a number of the studies, assisted by ASL. All authors read and approved the final manuscript.
